# Investigation of early signs of peripheral artery disease in patients with schizophrenia using toe-brachial index

**DOI:** 10.1192/j.eurpsy.2021.2153

**Published:** 2021-08-13

**Authors:** L. Jørgensen, C. Tranekær Hostrup, S. Eggert Jensen, R. Ernst Nielsen

**Affiliations:** 1 Psychiatry, Aalborg University Hospital, Aalborg, Denmark; 2 Department Of Clinical Medicine, Aalborg University, Aalborg, Denmark; 3 Cardiology, Aalborg University Hospital, Aalborg, Denmark

**Keywords:** toe-brachial index, Mortality, atherosclerosis, Cardiology

## Abstract

**Introduction:**

Patients with schizophrenia have a reduced life expectancy compared to the general population, and cardiovascular diseases contribute to this. Peripheral arterial disease (PAD) is associated with excess all-cause mortality and specifically with cardiovascular morbidity and mortality. The risk factors for PAD, such as diabetes, smoking, hypertension, dyslipidaemia and obesity, are more common among patients with schizophrenia which could contribute to a possibly higher prevalence of PAD among patients with schizophrenia.

**Objectives:**

To investigate PAD utilizing toe brachial index (TBI) in a population of patients diagnosed with schizophrenia with the purpose of establishing prevalence rates amongst newly diagnosed as well as more chronic patients.

**Methods:**

A cross-sectional study of patients with schizophrenia (ICD10-diagnosis F20 or F25) with a study population of 57 patients diagnosed with schizophrenia within the last 2 years, psychiatric healthy controls matched by age, sex and smoking status and 142 patients with a schizophrenia diagnosis more than 10 years ago. The primary outcome is TBI in patients with schizophrenia stratified to the two subpopulations. The TBI will be calculated from the arm and toe systolic pressures. The toe pressures were measured using photoplethysmography (SysToe®, Atys Medical).

**Results:**

No results are available yet. The cohort will be described by age, sex, smoking status, body fat percentage and physical comorbidities. The TBI of the two subpopulations will be compared with psychiatrically healthy controls using paired t-tests if data is normally distributed. If transformation is unsuitable, Wilcoxon test will be carried out instead.

**Conclusions:**

No results are available yet. Results will be presented at the EPA’s congress 2021.

**Disclosure:**

No significant relationships.

